# Correction: Adverse events of androgen receptor pathway inhibitors in prostate cancer from real world data

**DOI:** 10.1371/journal.pone.0340507

**Published:** 2026-01-05

**Authors:** Jongsoo Lee, Miho Song, Subeen Leem, Jong-Yeup Kim, Sung Ryul Shim, Jae Heon Kim, Benjamin I. Chung

The fifth to seventh authors are listed out of order. Please view the correct author order, affiliations, and citation here:

Jongsoo Lee 1, Miho Song 2, Subeen Leem 3, Jong-Yeup Kim 3–5 Sung Ryul Shim 3,4, Jae Heon Kim 2, Benjamin I. Chung 6

**1** Department of Urology, Severance Hospital, Yonsei University College of Medicine, Seoul, Republic of Korea, **2** Department of Urology, Soonchunhyang University Seoul Hospital, Soonchunhyang University College of Medicine, Seoul, Republic of Korea, **3** Evidence Based Research Center, Konyang University Hospital, Daejeon, Republic of Korea, **4** Department of Biomedical Informatics, College of Medicine, Konyang University, Daejeon, Republic of Korea, **5** Department of Otorhinolaryngology-Head and Neck Surgery, College of Medicine, Konyang University, Daejeon, Republic of Korea, **6** Department of Urology, Stanford University Medical Center, Stanford, California, United States of America.

Lee J, Song M, Leem S, Kim J-Y, Shim SR, Kim JH et al. (2025) Adverse events of androgen receptor pathway inhibitors in prostate cancer from real world data. PLoS One 20(10): e0335459. https://doi.org/10.1371/journal.pone.0335459.
