# Supplementary material for: Adverse events of androgen receptor pathway inhibitors in prostate cancer from real world data
Source: PLoS One. 2025 Oct 24;20(10):e0335459. doi: 10.1371/journal.pone.0335459 (PMC12551900; doi:10.1371/journal.pone.0335459)
Supplement: S1 Table — (PDF) [file pone.0335459.s001.pdf]

Supplemental Table S1. The adverse events of prostate cancer medication were based on Medical Dictionary for Regulatory Activities (MedDRA) concepts at the preferred term (PT) level

We considered the following PTs as related to prostate cancer medication AEs. Representative three AEs for each disease group were indicated: 'death [10011906]', 'drug ineffective [10013709]', 'malignant neoplasm progression [10051398]' were grouped into "Lack of efficacy"; 'fatigue [10016256]', 'asthenia [10003549]', 'decreased appetite [10061428]' were grouped into "General complications"; 'infection [10021789]', 'chills [10008531]', 'influenza [10022000]' were grouped into "Infection"; 'dementia [10012267]', 'insomnia [10022437]', 'depression [10012378]' were grouped into "Central Nervous System (CNS)"; 'sinusitis [10040753]', 'visual impairment [10047571]', 'dysphonia [10013952]' were grouped into "OPH/ENT"; 'dyspnoea [10013968]', 'pneumonia [10035664]', 'cough [10011224]' were grouped into "Respiratory"; 'myalgia [10028411]', 'back pain [10003988]', 'musculoskeletal stiffness [10052904]' were grouped into "Musculoskeletal"; 'hypertension [10020772]', 'anaemia [10002034]', 'blood pressure increased [10005750]' were grouped into "Vascular"; 'blood glucose increased [10005557]', 'diabetes mellitus [10012601]', 'hyperglycaemia [10020635]' were grouped into "Endocrine"; 'nausea [10028813]', 'diarrhoea [10012735]', 'constipation [10010774]' were grouped into "Gastro intestinal"; 'haematuria [10018867]', 'nephrolithiasis [10029148]', 'urinary incontinence [10046543]' were grouped into "Kidney/Urology"; 'rash [10037844]', 'pruritus [10037087]', 'erythema [10015150]' were grouped into "Skin".
