# Supplementary material for: Adverse events of androgen receptor pathway inhibitors in prostate cancer from real world data
Source: PLoS One. 2025 Oct 24;20(10):e0335459. doi: 10.1371/journal.pone.0335459 (PMC12551900; doi:10.1371/journal.pone.0335459)
Supplement: S2 Table — (PDF) [file pone.0335459.s002.pdf]

Supplemental Table S2. Proportional reporting ratios in Group 1 to Group 5

**Symptoms**	Specific AEs of Group 1 to 5 only	All AEs of Group 1 to 5 only	Specific AEs of All treatments	All AE of All treatments	PRR	95% CIL	95% CIH
Lack of efficacy	18,215	107,582	31,847	220,064	1.397	1.369	1.426
General complications	13,399	107,582	22,050	220,064	1.619	1.578	1.661
Infection	2,794	107,582	4,075	220,064	2.280	2.136	2.435
CNS	9,900	107,582	15,640	220,064	1.803	1.748	1.861
OPH/ENT	3,226	107,582	5,222	220,064	1.690	1.599	1.786
Respiratory	4,198	107,582	6,234	220,064	2.156	2.046	2.271
Musculoskeletal	9,710	107,582	15,072	220,064	1.893	1.833	1.955
Vascular	10,638	107,582	16,225	220,064	1.991	1.930	2.054
Endocrine	3,146	107,582	5,474	220,064	1.413	1.340	1.490
Gastro intestinal	11,900	107,582	18,962	220,064	1.762	1.713	1.812
Kidney/Urology	5,027	107,582	7,400	220,064	2.215	2.111	2.324
Skin	5,236	107,582	8,287	220,064	1.794	1.717	1.875
Others	10,193	107,582	18,688	220,064	1.255	1.221	1.289

Note: Data are from US FDA's Adverse Event Reporting System (FAERS) through to April 30, 2024. Group 1, Enzalutamide with other medications (excluding other ARPIs); Group 2, Apalutamide with other medications (excluding other ARPIs); Group 3, Darolutamide with other medications (excluding other ARPIs); Group 4, Abiraterone with other medications (excluding other ARPIs); Group 5, Abiraterone + Enzalutamide with other medications (excluding Apalutamide or Darolutamide). PRR, proportional reporting ratio. Missing values removed. Allow more than one adverse events calculation per patient.
