# Supplementary material for: Adverse events of androgen receptor pathway inhibitors in prostate cancer from real world data
Source: PLoS One. 2025 Oct 24;20(10):e0335459. doi: 10.1371/journal.pone.0335459 (PMC12551900; doi:10.1371/journal.pone.0335459)
Supplement: S3 Table — (PDF) [file pone.0335459.s003.pdf]

Supplemental Table S3. Proportional reporting ratios in Group 1

**Symptoms**	Specific AEs of Group 1 only	All AEs of Group 1 only	Specific AEs of All treatments	All AE of All treatments	PRR	95% CIL	95% CIH
Lack of efficacy	12,251	73,139	31,847	220,064	1.256	1.230	1.282
General complications	10,329	73,139	22,050	220,064	1.770	1.727	1.815
Infection	1,518	73,139	4,075	220,064	1.193	1.120	1.270
CNS	7,677	73,139	15,640	220,064	1.937	1.880	1.996
OPH/ENT	2,574	73,139	5,222	220,064	1.953	1.851	2.060
Respiratory	2,485	73,139	6,234	220,064	1.332	1.267	1.400
Musculoskeletal	7,103	73,139	15,072	220,064	1.791	1.737	1.846
Vascular	6,573	73,139	16,225	220,064	1.368	1.328	1.410
Endocrine	2,039	73,139	5,474	220,064	1.192	1.130	1.259
Gastro intestinal	8,339	73,139	18,962	220,064	1.577	1.535	1.620
Kidney/Urology	2,736	73,139	7,400	220,064	1.178	1.125	1.234
Skin	3,487	73,139	8,287	220,064	1.459	1.398	1.523
Others	6,028	73,139	18,688	220,064	0.957	0.929	0.985

Note: Data are from US FDA's Adverse Event Reporting System (FAERS) through to April 30, 2024 Group 1, Enzalutamide with other medications (excluding other ARPIs); Group 2, Apalutamide with other medications (excluding other ARPIs); Group 3, Darolutamide with other medications (excluding other ARPIs); Group 4, Abiraterone with other medications (excluding other ARPIs); Group 5, Abiraterone + Enzalutamide with other medications (excluding Apalutamide or Darolutamide). PRR, proportional reporting ratio. Missing values removed. Allow more than one adverse events calculation per patient.
