# Supplementary material for: Adverse events of androgen receptor pathway inhibitors in prostate cancer from real world data
Source: PLoS One. 2025 Oct 24;20(10):e0335459. doi: 10.1371/journal.pone.0335459 (PMC12551900; doi:10.1371/journal.pone.0335459)
Supplement: S4 Table — (PDF) [file pone.0335459.s004.pdf]

Supplemental Table S4. Proportional reporting ratios in Group 2

**Symptoms**	Specific AEs of Group 2 only	All AEs of Group 2 only	Specific AEs of All treatments	All AE of All treatments	PRR	95% CIL	95% CIH
Lack of efficacy	783	6,209	31,847	220,064	0.868	0.813	0.927
General complications	678	6,209	22,050	220,064	1.093	1.017	1.174
Infection	217	6,209	4,075	220,064	1.937	1.694	2.216
CNS	496	6,209	15,640	220,064	1.128	1.036	1.229
OPH/ENT	171	6,209	5,222	220,064	1.166	1.003	1.355
Respiratory	239	6,209	6,234	220,064	1.373	1.210	1.559
Musculoskeletal	450	6,209	15,072	220,064	1.060	0.969	1.160
Vascular	682	6,209	16,225	220,064	1.511	1.406	1.624
Endocrine	258	6,209	5,474	220,064	1.704	1.507	1.925
Gastro intestinal	523	6,209	18,962	220,064	0.977	0.899	1.061
Kidney/Urology	270	6,209	7,400	220,064	1.304	1.158	1.469
Skin	776	6,209	8,287	220,064	3.558	3.322	3.812
Others	666	6,209	18,688	220,064	1.273	1.184	1.369

Note: Data are from US FDA's Adverse Event Reporting System (FAERS) through to April 30, 2024. Group 1, Enzalutamide with other medications (excluding other ARPIs); Group 2, Apalutamide with other medications (excluding other ARPIs); Group 3, Darolutamide with other medications (excluding other ARPIs); Group 4, Abiraterone with other medications (excluding other ARPIs); Group 5, Abiraterone + Enzalutamide with other medications (excluding Apalutamide or Darolutamide). PRR, proportional reporting ratio. Missing values removed. Allow more than one adverse events calculation per patient.
