# Supplementary material for: Adverse events of androgen receptor pathway inhibitors in prostate cancer from real world data
Source: PLoS One. 2025 Oct 24;20(10):e0335459. doi: 10.1371/journal.pone.0335459 (PMC12551900; doi:10.1371/journal.pone.0335459)
Supplement: S5 Table — (PDF) [file pone.0335459.s005.pdf]

Supplemental Table S5. Proportional reporting ratios in Group 3

**Symptoms**	Specific AEs of Group 3 only	All AEs of Group 3 only	Specific AEs of All treatments	All AE of All treatments	PRR	95% CIL	95% CIH
Lack of efficacy	138	1,825	31,847	220,064	0.520	0.443	0.611
General complications	225	1,825	22,050	220,064	1.233	1.091	1.392
Infection	57	1,825	4,075	220,064	1.696	1.312	2.194
CNS	162	1,825	15,640	220,064	1.252	1.080	1.450
OPH/ENT	39	1,825	5,222	220,064	0.900	0.659	1.229
Respiratory	82	1,825	6,234	220,064	1.594	1.288	1.972
Musculoskeletal	183	1,825	15,072	220,064	1.470	1.281	1.686
Vascular	189	1,825	16,225	220,064	1.409	1.232	1.613
Endocrine	51	1,825	5,474	220,064	1.125	0.857	1.476
Gastro intestinal	193	1,825	18,962	220,064	1.230	1.076	1.405
Kidney/Urology	111	1,825	7,400	220,064	1.821	1.519	2.183
Skin	139	1,825	8,287	220,064	2.040	1.737	2.396
Others	256	1,825	18,688	220,064	1.661	1.484	1.859

Note: Data are from US FDA's Adverse Event Reporting System (FAERS) through to April 30, 2024. Group 1, Enzalutamide with other medications (excluding other ARPIs); Group 2, Apalutamide with other medications (excluding other ARPIs); Group 3, Darolutamide with other medications (excluding other ARPIs); Group 4, Abiraterone with other medications (excluding other ARPIs); Group 5, Abiraterone + Enzalutamide with other medications (excluding Apalutamide or Darolutamide). PRR, proportional reporting ratio. Missing values removed. Allow more than one adverse events calculation per patient.
