# Supplementary material for: Adverse events of androgen receptor pathway inhibitors in prostate cancer from real world data
Source: PLoS One. 2025 Oct 24;20(10):e0335459. doi: 10.1371/journal.pone.0335459 (PMC12551900; doi:10.1371/journal.pone.0335459)
Supplement: S6 Table — (PDF) [file pone.0335459.s006.pdf]

Supplemental Table S6. Proportional reporting ratios in Group 4

**Symptoms**	Specific AEs of Group 4 only	All AEs of Group 4 only	Specific AEs of All treatments	All AE of All treatments	PRR	95% CIL	95% CIH
Lack of efficacy	4,720	24,476	31,847	220,064	1.390	1.353	1.429
General complications	1,977	24,476	22,050	220,064	0.787	0.753	0.823
Infection	929	24,476	4,075	220,064	2.360	2.196	2.536
CNS	1,403	24,476	15,640	220,064	0.787	0.747	0.830
OPH/ENT	412	24,476	5,222	220,064	0.684	0.620	0.756
Respiratory	1,274	24,476	6,234	220,064	2.053	1.933	2.180
Musculoskeletal	1,774	24,476	15,072	220,064	1.066	1.016	1.118
Vascular	2,946	24,476	16,225	220,064	1.773	1.708	1.840
Endocrine	754	24,476	5,474	220,064	1.277	1.183	1.377
Gastro intestinal	2,633	24,476	18,962	220,064	1.289	1.240	1.339
Kidney/Urology	1,765	24,476	7,400	220,064	2.503	2.377	2.636
Skin	763	24,476	8,287	220,064	0.810	0.753	0.872
Others	3,126	24,476	18,688	220,064	1.605	1.549	1.664

Note: Data are from US FDA's Adverse Event Reporting System (FAERS) through to April 30, 2024. Group 1, Enzalutamide with other medications (excluding other ARPIs); Group 2, Apalutamide with other medications (excluding other ARPIs); Group 3, Darolutamide with other medications (excluding other ARPIs); Group 4, Abiraterone with other medications (excluding other ARPIs); Group 5, Abiraterone + Enzalutamide with other medications (excluding Apalutamide or Darolutamide). PRR, proportional reporting ratio. Missing values removed. Allow more than one adverse events calculation per patient.
