# Supplementary material for: Adverse events of androgen receptor pathway inhibitors in prostate cancer from real world data
Source: PLoS One. 2025 Oct 24;20(10):e0335459. doi: 10.1371/journal.pone.0335459 (PMC12551900; doi:10.1371/journal.pone.0335459)
Supplement: S7 Table — (PDF) [file pone.0335459.s007.pdf]

Supplemental Table S7. Proportional reporting ratios in Group 5

**Symptoms**	Specific AEs of Group 5 only	All AEs of Group 5 only	Specific AEs of All treatments	All AE of All treatments	PRR	95% CIL	95% CIH
Lack of efficacy	323	1,933	31,847	220,064	1.156	1.048	1.275
General complications	190	1,933	22,050	220,064	0.981	0.857	1.122
Infection	73	1,933	4,075	220,064	2.058	1.640	2.583
CNS	162	1,933	15,640	220,064	1.181	1.019	1.369
OPH/ENT	30	1,933	5,222	220,064	0.652	0.457	0.931
Respiratory	118	1,933	6,234	220,064	2.177	1.825	2.597
Musculoskeletal	200	1,933	15,072	220,064	1.518	1.331	1.730
Vascular	248	1,933	16,225	220,064	1.752	1.560	1.967
Endocrine	44	1,933	5,474	220,064	0.914	0.682	1.226
Gastro intestinal	212	1,933	18,962	220,064	1.276	1.124	1.448
Kidney/Urology	145	1,933	7,400	220,064	2.255	1.926	2.641
Skin	71	1,933	8,287	220,064	0.975	0.775	1.226
Others	117	1,933	18,688	220,064	0.711	0.596	0.848

Note: Data are from US FDA's Adverse Event Reporting System (FAERS) through to April 30, 2024. Group 1, Enzalutamide with other medications (excluding other ARPIs); Group 2, Apalutamide with other medications (excluding other ARPIs); Group 3, Darolutamide with other medications (excluding other ARPIs); Group 4, Abiraterone with other medications (excluding other ARPIs); Group 5, Abiraterone + Enzalutamide with other medications (excluding Apalutamide or Darolutamide). PRR, proportional reporting ratio. Missing values removed. Allow more than one adverse events calculation per patient.
