# Supplementary material for: Adverse events of androgen receptor pathway inhibitors in prostate cancer from real world data
Source: PLoS One. 2025 Oct 24;20(10):e0335459. doi: 10.1371/journal.pone.0335459 (PMC12551900; doi:10.1371/journal.pone.0335459)
Supplement: Attachment — Submitted filename: comments 14 (2).docx [file pone.0335459.s009.docx]

This study provides a valuable contribution to the field of prostate cancer treatment by analyzing real-world data (RWD) from the FAERS database to evaluate the adverse events (AEs) associated with androgen receptor pathway inhibitors (ARPIs). The large sample size and comprehensive analysis of AEs across different ARPI groups offer important insights into their safety profiles. The use of proportional reporting ratios (PRRs) to quantify AE risks is methodologically sound, and the findings highlight the need for individualized treatment decisions based on AE profiles. The study addresses a critical gap in the literature by leveraging RWD to complement clinical trial evidence, making it highly relevant for clinicians and researchers. The manuscript is promising but requires revisions to address these issues before acceptance.

**Comments for Revision:**

1. The abstract mentions data collection from May 30, 2014, to May 30, 2024, but the results section states April 30, 2014, to April 30, 2024. This discrepancy needs correction for consistency.
2. The term "ANY" in the treatment groups (e.g., Enzalutamide+ANY) is ambiguous. Specify whether it refers to concomitant medications, other ARPIs, or additional therapies to avoid confusion.
3. The rationale for excluding cases with unknown age or missing data is not provided. Explain how this exclusion might impact the representativeness of the results.
4. The study does not account for potential confounders such as comorbidities, concurrent medications, or disease stage. Discuss how these factors might influence AE reporting and interpretations.
5. Provide more details on the statistical methods used, such as how PRRs were calculated and adjusted for multiple comparisons. Clarify whether any corrections (e.g., Bonferroni) were applied to mitigate false positives.
6. The passive and voluntary nature of FAERS reporting can lead to underreporting or bias. Elaborate on how these limitations might affect the study's conclusions and suggest ways to mitigate them.
7. While PRRs highlight statistical associations, their clinical significance is unclear. Discuss whether the observed PRRs translate to meaningful differences in patient outcomes or treatment decisions.
8. The criteria for grouping AEs into 12 categories (e.g., "vascular," "CNS") are not detailed. Provide a clear methodology or reference for how these groupings were determined.
9. The study compares ARPIs indirectly but lacks direct head-to-head comparisons. Acknowledge this limitation and discuss how it affects the interpretation of relative safety.
10. Although IRB approval was waived, briefly discuss ethical implications of using publicly available data without patient consent, especially regarding privacy and data anonymization.
