# Supplementary material for: Adverse events of androgen receptor pathway inhibitors in prostate cancer from real world data
Source: PLoS One. 2025 Oct 24;20(10):e0335459. doi: 10.1371/journal.pone.0335459 (PMC12551900; doi:10.1371/journal.pone.0335459)
Supplement: Attachment — Submitted filename: Response to Reviewers 250701.docx [file pone.0335459.s011.docx]

Dear Editor and Reviewers

We sincerely thank the editor and reviewers for their thoughtful and valuable comments.

The authors have answered the reviewers' questions to the best of our ability and hope that this study will be a step forward in the scientific understanding of the adverse effects of ARPIs in prostate cancer with RWD.

The reviewer's questions are in black and the authors' answers are in blue.

PONE-D-25-09399

Adverse events of androgen receptor pathway inhibitors in prostate cancer from real world data

PLOS ONE

Dear Dr. SHIM,

Thank you for submitting your manuscript to PLOS ONE. After careful consideration, we feel that it has merit but does not fully meet PLOS ONE’s publication criteria as it currently stands. Therefore, we invite you to submit a revised version of the manuscript that addresses the points raised during the review process.

Please submit your revised manuscript by Jun 19 2025 11:59PM. If you will need more time than this to complete your revisions, please reply to this message or contact the journal office at plosone@plos.org. When you're ready to submit your revision, log on to https://www.editorialmanager.com/pone/ and select the 'Submissions Needing Revision' folder to locate your manuscript file.

Please include the following items when submitting your revised manuscript:

A rebuttal letter that responds to each point raised by the academic editor and reviewer(s). You should upload this letter as a separate file labeled 'Response to Reviewers'.

A marked-up copy of your manuscript that highlights changes made to the original version. You should upload this as a separate file labeled 'Revised Manuscript with Track Changes'.

An unmarked version of your revised paper without tracked changes. You should upload this as a separate file labeled 'Manuscript'.

If you would like to make changes to your financial disclosure, please include your updated statement in your cover letter. Guidelines for resubmitting your figure files are available below the reviewer comments at the end of this letter.

If applicable, we recommend that you deposit your laboratory protocols in protocols.io to enhance the reproducibility of your results. Protocols.io assigns your protocol its own identifier (DOI) so that it can be cited independently in the future. For instructions see: https://journals.plos.org/plosone/s/submission-guidelines#loc-laboratory-protocols. Additionally, PLOS ONE offers an option for publishing peer-reviewed Lab Protocol articles, which describe protocols hosted on protocols.io. Read more information on sharing protocols at https://plos.org/protocols?utm_medium=editorial-email&utm_source=authorletters&utm_campaign=protocols.

We look forward to receiving your revised manuscript.

Kind regards,

Xing-Xiong An, M.D.

Academic Editor

PLOS ONE

Journal Requirements:

When submitting your revision, we need you to address these additional requirements.

1. Please ensure that your manuscript meets PLOS ONE's style requirements, including those for file naming. The PLOS ONE style templates can be found at

https://journals.plos.org/plosone/s/file?id=wjVg/PLOSOne_formatting_sample_main_body.pdf and

https://journals.plos.org/plosone/s/file?id=ba62/PLOSOne_formatting_sample_title_authors_affiliations.pdf

2. Thank you for stating the following financial disclosure: [This work was supported by Soonchunhyang University Research Fund. National Research Foundation of Korea (NRF) grant funded by the Korea government (MSIT) (2022R1A2C3005586)].

Please state what role the funders took in the study. If the funders had no role, please state: ""The funders had no role in study design, data collection and analysis, decision to publish, or preparation of the manuscript.""

If this statement is not correct you must amend it as needed.

Please include this amended Role of Funder statement in your cover letter; we will change the online submission form on your behalf.

3. Thank you for uploading your study's underlying data set. Unfortunately, the repository you have noted in your Data Availability statement does not qualify as an acceptable data repository according to PLOS's standards.

At this time, please upload the minimal data set necessary to replicate your study's findings to a stable, public repository (such as figshare or Dryad) and provide us with the relevant URLs, DOIs, or accession numbers that may be used to access these data. For a list of recommended repositories and additional information on PLOS standards for data deposition, please see https://journals.plos.org/plosone/s/recommended-repositories.

[Note: HTML markup is below. Please do not edit.]

Reviewers' comments:

Reviewer's Responses to Questions

Comments to the Author

1. Is the manuscript technically sound, and do the data support the conclusions?

The manuscript must describe a technically sound piece of scientific research with data that supports the conclusions. Experiments must have been conducted rigorously, with appropriate controls, replication, and sample sizes. The conclusions must be drawn appropriately based on the data presented.

Reviewer #1: Yes

Reviewer #2: Yes

Reviewer #3: Yes

Reviewer #4: Yes

2. Has the statistical analysis been performed appropriately and rigorously?

Reviewer #1: No

Reviewer #2: I Don't Know

Reviewer #3: Yes

Reviewer #4: Yes

3. Have the authors made all data underlying the findings in their manuscript fully available?

The PLOS Data policy requires authors to make all data underlying the findings described in their manuscript fully available without restriction, with rare exception (please refer to the Data Availability Statement in the manuscript PDF file). The data should be provided as part of the manuscript or its supporting information, or deposited to a public repository. For example, in addition to summary statistics, the data points behind means, medians and variance measures should be available. If there are restrictions on publicly sharing data—e.g. participant privacy or use of data from a third party—those must be specified.

Reviewer #1: Yes

Reviewer #2: Yes

Reviewer #3: Yes

Reviewer #4: Yes

4. Is the manuscript presented in an intelligible fashion and written in standard English?

PLOS ONE does not copyedit accepted manuscripts, so the language in submitted articles must be clear, correct, and unambiguous. Any typographical or grammatical errors should be corrected at revision, so please note any specific errors here.

Reviewer #1: Yes

Reviewer #2: No

Reviewer #3: Yes

Reviewer #4: Yes

5. Review Comments to the Author

Please use the space provided to explain your answers to the questions above. You may also include additional comments for the author, including concerns about dual publication, research ethics, or publication ethics. (Please upload your review as an attachment if it exceeds 20,000 characters)

Reviewer #1: Interesting topic.

By addressing these points, the study will be clearer, more rigorous, and clinically relevant.

Q1-1. Context on ARPI Side Effects

You would better cite recent clinical meta-analyses (e.g., PMIDs: 39223303, 38097723) to address the evidence gap on ARPI side effects.

Highlight the need for real-world data and consider discussing why different ARPIs have varied side effect profiles.

A1-1) We sincerely appreciate your valuable comments regarding the new insight. Based on your recommendation, we have added the consideration in maain body as below (line 243-248).

" Recent met-analysis study showed that combined treatments result in better survival than does ADT alone (Br J Cancer. 2024 Nov;131(8):1363-1377). About the side effects of ARPI, one recent meta-analysis showed that darolutamide had a toxicity profile comparable to placebo with the exception of bone fractures. In the absence of head-to-head comparison studies between the different ARPIs, the results of this meta-analysis suggested a preferred use of darolutamide in the approved settings (Prostate Cancer Prostatic Dis . 2024 Sep;27(3):385-392).”

Q1-2. Treatment Group Rationale

Please clarify the logic behind grouping ARPIs for a clearer understanding, especially for non-specialist readers. Expand the Methods section to better explain groupings and how they reflect real-world prescribing practices.

A1-2) We sincerely appreciate your valuable comments regarding the treatment group rationale. The combinations are doublet or triplet treatment combinations by drug. In other words, you can think of it as a combination of doublet or triplet including ADT. For better understanding, I have included the following (line 166-167):

“Each group by drug was doublet or triplet treatment based on ADT”

Q1-3. Attribution of Side Effects to Specific ARPIs

You have mentioned that combination therapies make it hard to attribute side effects to a single drug.

Suggest further analysis to separate combination treatments and emphasize that PRRs can't establish causality.

A1-3) We sincerely appreciate your valuable comments regarding the clarity and presentation of our findings. Combination therapy corresponds to group 5 (Abiraterone + Enzalutamide) only, and the rest all represent individual ARPI treatment. Based on your recommendation, we have correctly designated this "ANY" group in main body (footnotes of tables and figures also) and supplement document as below.

“Group 1, Enzalutamide with other medications (excluding other ARPIs); Group 2, Apalutamide with other medications (excluding other ARPIs); Group 3, Darolutamide with other medications (excluding other ARPIs); Group 4, Abiraterone with other medications (excluding other ARPIs); Group 5, Abiraterone + Enzalutamide with other medications (excluding Apalutamide or Darolutamide).”

Steroids widely used in general chemotherapy other than ARPI were excluded because they were not the main treatments of interest in this study. This study is a cross-sectional study, so it is not easy to find causality. Also, there are various drug combinations, so it is not easy to create all the combinations. However, this study focuses on analyzing the side effects of specific ALPIs that have recently become a problem (skin problems, lethargy, etc.).

Q1-4. Generalizability and Regional Limitations

Acknowledge that while flutamide is FDA-approved, it was not included in the study. This exclusion may limit the generalizability and the findings may not apply globally, particularly for regions still using non-FDA-approve or other older therapies.

A1-4) We sincerely appreciate your valuable comments. First- or second-generation androgen receptor blockers such as flutamide or bicalutamide are not recommended for use in the guideline either. They are not included in the ALPI, so it seems reasonable to exclude them.

Q1-5. Confounder Adjustment

Adjust for potential confounders (e.g., age, comorbidities) to ensure accurate results.

A1-5) We sincerely appreciate your valuable comments regarding the interpretation of the potential confounders. The inability to control for potential confounders limits its applicability to real-world practice. Unfortunately, FAERS does not provide detailed exact covariates (comorbidities, concurrent medications, disease stage, etc.) for individual participants, so we tried to control for these as much as possible through stratification by age, gender, and treatment group. The findings should therefore be interpreted as hypothesis-generating and descriptive of reporting trends, rather than definitive comparative risk quantifications as it is primarily stemming from the FAERS data.

Based on your recommendation, we have added this limitation in main body as below (line 332-336).

" Third, the inability to control for potential confounders limits its applicability to real-world practice. Unfortunately, FAERS does not provide detailed exact covariates (comorbidities, concurrent medications, disease stage, etc.) for individual participants, so we tried to control for these as much as possible through stratification by age, gender, and treatment group."

Q1-6. Reporting Biases in FAERS

Address biases in FAERS data (underreporting, overreporting) and how they impact the results.

A1-6) We sincerely appreciate your valuable comments regarding the interpretation of the underreporting and overreporting. Underreporting and overreporting are important issues in the study. Even in this FAERS data, there is a significant number of participants who did not report their age and gender, which appears to be a significant bias. However, FAERS is a large big data set and we believe that the PRR for each of the individual groups of ARPI (Enzalutamide, Apalutamide, Darolutamide, Abiraterone, and Abiraterone with Enzalutamide), which is our primary interest, is likely to remain constant with a constant effect size because of the large number of missing participants. In addition, we can assume that the missingness also occurred in all 12 disease groups in this study, so it is judged to be a non-differential misclassification error, and the direction is not always towards the null value. Therefore, we can maintain confidence in this study.

Based on your recommendation, we have added this limitation in main body as below (line 324-332).

“Second, even in this FAERS data, there is a significant number of participants who did not report their age and gender, which appears to be a significant bias. However, FAERS is a large big data set and we believe that the PRR for each of the individual groups of ARPI (Enzalutamide, Apalutamide, Darolutamide, Abiraterone, and Abiraterone with Enzalutamide), which is our primary interest, is likely to remain constant with a constant effect size because of the large number of missing participants. In addition, we can assume that the missingness also occurred in all 12 disease groups in this study, so it is judged to be a non-differential misclassification error, and the direction is not always towards the null value. Therefore, we can maintain confidence in this study."

Q1-7. Temporal Bias

Consider the shorter reporting window for newer drugs like darolutamide, which could skew AE comparisons.

A1-7) We sincerely appreciate your exact comments regarding darolutamide due to the short observation period. Based on your recommendation, we have added this limitation in main body as below (line 283-285).

“In addition, due to the short observation period, darolutamide must be careful in interpreting the side effects involved, and more accumulated data must be completed and then reanalyzed.”

Q1-8. Clinical Actionability

Suggest how clinicians might use the findings in practice, such as avoiding apalutamide for patients with skin issues.

A1-8) We sincerely appreciate your valuable comments. Based on our results, it is possible to accurately educate patients about the possibility of skin problems before prescribing a specific ALPI, namely apalutamide, and to enable early response and dose adjustment when they occur. In addition, the high side effects of other specific ALPIs can be clinically applied in the same

Q1-9. Sample Size Concerns

Note that smaller groups (e.g., Group 3, n=733) may result in less stable PRR estimates. Consider confidence interval overlap in comparisons.

A1-9) We sincerely appreciate your exact comments regarding darolutamide due to the short observation period and small sample size.

Based on your recommendation, we have added this limitation in main body as below (line 283-285).

“In addition, due to the short observation period, darolutamide must be careful in interpreting the side effects involved, and more accumulated data must be completed and then reanalyzed.”

Please check supplement Table S5, you can find the detailed PRR and CIs for each disease group.

Q1-10. Language and Clarity

Recommend improving clarity and correcting grammar (e.g., "instalement" to "implementation").

A1-10) We sincerely appreciate your exact comments. We check it again regarding the clarity and presentation of our findings. This study was supervised by a urologist at Stanford University in the United States, who is a native English speaker (Benjamin I. Chung).

Reviewer #2: This study provides a valuable contribution to the field of prostate cancer treatment by analyzing real-world data (RWD) from the FAERS database to evaluate the adverse events (AEs) associated with androgen receptor pathway inhibitors (ARPIs). The large sample size and comprehensive analysis of AEs across different ARPI groups offer important insights into their safety profiles. The use of proportional reporting ratios (PRRs) to quantify AE risks is methodologically sound, and the findings highlight the need for individualized treatment decisions based on AE profiles. The study addresses a critical gap in the literature by leveraging RWD to complement clinical trial evidence, making it highly relevant for clinicians and researchers. The manuscript is promising but requires revisions to address these issues before acceptance.

Comments for Revision:

Q2-1. The abstract mentions data collection from May 30, 2014, to May 30, 2024, but the results section states April 30, 2014, to April 30, 2024. This discrepancy needs correction for consistency.

A2-1) We sincerely appreciate your exact comments regarding the methodological transparency of our data processing. We corrected it to April in main body (footnotes of tables and figures also) and supplement document.

Q2-2. The term "ANY" in the treatment groups (e.g., Enzalutamide+ANY) is ambiguous. Specify whether it refers to concomitant medications, other ARPIs, or additional therapies to avoid confusion.

A2-2) We sincerely appreciate your valuable comments regarding the clarity and presentation of our findings. Based on your recommendation, we have correctly designated this "ANY" group in main body (footnotes of tables and figures also) and supplement document as below.

“Group 1, Enzalutamide with other medications (excluding other ARPIs); Group 2, Apalutamide with other medications (excluding other ARPIs); Group 3, Darolutamide with other medications (excluding other ARPIs); Group 4, Abiraterone with other medications (excluding other ARPIs); Group 5, Abiraterone + Enzalutamide with other medications (excluding Apalutamide or Darolutamide).”

Q2-3. The rationale for excluding cases with unknown age or missing data is not provided. Explain how this exclusion might impact the representativeness of the results.

A2-3) We sincerely appreciate your valuable comments regarding the interpretation of the missing data. The missing data is an important issue in the study. Even in this FAERS data, there is a significant number of participants who did not report their age and gender, which appears to be a significant bias. However, FAERS is a large big data set and we believe that the PRR for each of the individual groups of ARPI (Enzalutamide, Apalutamide, Darolutamide, Abiraterone, and Abiraterone with Enzalutamide), which is our primary interest, is likely to remain constant with a constant effect size because of the large number of missing participants. In addition, we can assume that the missingness also occurred in all 12 disease groups in this study, so it is judged to be a non-differential misclassification error, and the direction is not always towards the null value. Therefore, we can maintain confidence in this study.

Based on your recommendation, we have added this limitation in main body as below (line 324-332).

“Second, even in this FAERS data, there is a significant number of participants who did not report their age and gender, which appears to be a significant bias. However, FAERS is a large big data set and we believe that the PRR for each of the individual groups of ARPI (Enzalutamide, Apalutamide, Darolutamide, Abiraterone, and Abiraterone with Enzalutamide), which is our primary interest, is likely to remain constant with a constant effect size because of the large number of missing participants. In addition, we can assume that the missingness also occurred in all 12 disease groups in this study, so it is judged to be a non-differential misclassification error, and the direction is not always towards the null value. Therefore, we can maintain confidence in this study."

Q2-4. The study does not account for potential confounders such as comorbidities, concurrent medications, or disease stage. Discuss how these factors might influence AE reporting and interpretations.

A2-4) We sincerely appreciate your valuable comments regarding the interpretation of the potential confounders. The inability to control for potential confounders limits its applicability to real-world practice. Unfortunately, FAERS does not provide detailed exact covariates (comorbidities, concurrent medications, disease stage, etc.) for individual participants, so we tried to control for these as much as possible through stratification by age, gender, and treatment group. The findings should therefore be interpreted as hypothesis-generating and descriptive of reporting trends, rather than definitive comparative risk quantifications as it is primarily stemming from the FAERS data.

Based on your recommendation, we have added this limitation in main body as below (line 332-336).

" Third, the inability to control for potential confounders limits its applicability to real-world practice. Unfortunately, FAERS does not provide detailed exact covariates (comorbidities, concurrent medications, disease stage, etc.) for individual participants, so we tried to control for these as much as possible through stratification by age, gender, and treatment group."

Q2-5. Provide more details on the statistical methods used, such as how PRRs were calculated and adjusted for multiple comparisons. Clarify whether any corrections (e.g., Bonferroni) were applied to mitigate false positives.

A2-5) We sincerely appreciate your exact comments regarding the statistical methods we used. The proportional reporting ratio (PRR) is a widely employed approach for evaluating the significance of AEs. We also described it specifically with the calculation formula in line 167 - 186. The PRR quantifies the occurrence of events in the population of interest and the overall population. For multiple comparisons between groups, please see the supplementary document. We calculated PRRs for each of the 12 disease groups of interest and also stratified them within the individual groups of ARPIs (Enzalutamide, Apalutamide, Darolutamide, Abiraterone, Abiraterone with Enzalutamide) and analyzed all of them. We also stratified them by AE or ARPI so that clinicians can see how they might treat patients in the real world.

Q2-6. The passive and voluntary nature of FAERS reporting can lead to underreporting or bias. Elaborate on how these limitations might affect the study's conclusions and suggest ways to mitigate them.

A2-6) We sincerely appreciate your valuable comments regarding the data acquisition in FAERS. This comment appears to be related to the third question(Q2-3), data missing. The answer to this question is the same as the answer to the third question, Non-differential misclassification error, the direction is not always towards the null value.

Q2-7. While PRRs highlight statistical associations, their clinical significance is unclear. Discuss whether the observed PRRs translate to meaningful differences in patient outcomes or treatment decisions.

A2-7) We sincerely appreciate your valuable comments. Based on our results, it is possible to accurately educate patients about the possibility of skin problems before prescribing a specific ALPI, namely apalutamide, and to enable early response and dose adjustment when they occur. In addition, the high side effects of other specific ALPIs can be clinically applied in the same

Q2-8. The criteria for grouping AEs into 12 categories (e.g., "vascular," "CNS") are not detailed. Provide a clear methodology or reference for how these groupings were determined.

A2-8) We sincerely appreciate your valuable comments regarding the disease group categorization. The division into major adverse event groups is described in detail in the main body (lines 148-158). Also, please check supplement Table 1, you can find the detailed MeDRA concepts and PT level code for each disease group.

"Since the overall count of AE names were 4,440, which was too many, data preprocessing of all AEs into similar terms and related disease groups was necessary. Of all AE names, more than 100 reported ones were divided into 12 disease groups. According to data preprocessing, a total of 12 disease groups were based on MedDRA concepts at the PT level (Supplemental Table S1). Two researchers (SRS and JHK) independently reviewed the descriptions in the database to verify the accuracy of the adverse events related to prostate cancer medications AEs. One author (JHK), a specialist in Urology and prostate cancer; one author (SRS), a specialist in Epidemiology and Medical Informatics, validated the identified terms and their groupings and, validated the identified terms and their groupings. The authors also analyzed all narrative descriptions of concurrent illnesses and comorbidities in FAERS. In cases of disagreement regarding the descriptions, the final PTs were established through agreement among all investigators."

Q2-9. The study compares ARPIs indirectly but lacks direct head-to-head comparisons. Acknowledge this limitation and discuss how it affects the interpretation of relative safety.

A2-9) We sincerely appreciate your exact comments regarding the data comparison methods in FAERS. Early in our analysis, we considered the reviewer's methodology. Because it is not presented as individual exact data, but only as aggregate statistics, we established the analysis method while thinking about its use in real practice. Therefore, we first showed the incidence of AEs within individual ARPIs (Table 3) and secondly showed the frequency of adverse events of specific ARPIs in the entire population as PRR (Figure 2). As you mentioned, we could have made direct comparisons between ARPIs, but because the FAERS data does not provide precise covariates for individual patients, only aggregate statistics, we felt that analyzing incomplete observational data as a quantitative direct comparison between ARPIs would introduce more bias. Therefore, in the supplemental documents, we present the PRRs stratified by each ARPI group (Enzalutamide, Apalutamide, Darolutamide, Abiraterone, Abiraterone with Enzalutamide), and we have also categorized them by AE or ARPI so that clinicians can differentiate between them when treating patients.

Q2-10. Although IRB approval was waived, briefly discuss ethical implications of using publicly available data without patient consent, especially regarding privacy and data anonymization.

A2-10) We sincerely appreciate your kind comments regarding the research ethics. The ethics of the data are already described in 119-123 and in 141-145, and we understand that in the final stage of publication in Plos One, the Acknowledgments will also include a Data Availability and Ethics Statement.

“This study was exempted by the Institutional Review Board at Konyang University and Soonchunhyang University, as we utilized publicly available, anonymized data, satisfying the requirements for exemption as detailed in the Department of Health and Human Services regulations [18].”

“Information about the survey, including the questionnaires, methodology, and dataset description, can be found on the previously mentioned websites (https://www.fda.gov/drugs/surveillance/fda-adverse-event-reporting-system-faers).”

Reviewer #3: -In this study, the authors analyzed the adverse effects of prostate cancer medication agents (Enzalutamide, Apalutamide, Darolutamide, and Abiraterone) in prostate cancer patients from real-world data.

Q3-1. ARPIs are known to be effective in advanced prostate cancer treatments. It is important to evaluate the adverse events of treatments together with the patient and decide on the treatment option. Therefore, I think this type of research is valuable.

A3-1) We sincerely appreciate your valuable comments regarding the necessity of this study. The authors are always thinking about what the best treatment option for patients in the real practice is, and they are also trying to minimize the side effects they experience. We will continue to do more to help patients live and treat them. Thank you.

Q3-2. The adverse effects of treatments applied to patients can directly affect the quality of life. My suggestion to the authors; Literature data on the quality of life of patients receiving ARPI treatment can be briefly commented on in the discussion section.

A3-2) We sincerely appreciate your valuable comments. The side effects of ADT in the treatment of prostate cancer that has progressed so far are already widely known, but the side effects of the newly introduced ALPI are still not well known to both doctors and patients. According to recent studies, specific side effects of certain ALPIs are highly likely to significantly reduce the quality of life of patients, so through our study, it is possible to provide patients with advance education before prescribing a specific ALPI, recognize the occurrence of side effects in advance, and take early measures such as reducing the dose.

Reviewer #4: This manuscript is a retrospective observational study analyzing real-world adverse events (AEs) associated with androgen receptor pathway inhibitors (ARPIs) in prostate cancer patients using the FDA Adverse Event Reporting System (FAERS) database. The study provides valuable insights into the comparative safety profiles of different ARPIs, which can aid clinicians in personalized treatment decisions.

Q4-1. Is there anyway can the authors handle the missing data instead of exclufing them? please justify why did you remove them instead of missing value imputation technique.

A4-1) We sincerely appreciate your valuable comments regarding the interpretation of the missing data. The missing data is an important issue in the study. Even in this FAERS data, there is a significant number of participants who did not report their age and gender, which appears to be a significant bias. However, FAERS is a large big data set and we believe that the PRR for each of the individual groups of ARPI (Enzalutamide, Apalutamide, Darolutamide, Abiraterone, and Abiraterone with Enzalutamide), which is our primary interest, is likely to remain constant with a constant effect size because of the large number of missing participants. In addition, we can assume that the missingness also occurred in all 12 disease groups in this study, so it is judged to be a non-differential misclassification error, and the direction is not always towards the null value. Therefore, we can maintain confidence in this study.

Based on your recommendation, we have added this limitation in main body as below (line 324-332).

“Second, even in this FAERS data, there is a significant number of participants who did not report their age and gender, which appears to be a significant bias. However, FAERS is a large big data set and we believe that the PRR for each of the individual groups of ARPI (Enzalutamide, Apalutamide, Darolutamide, Abiraterone, and Abiraterone with Enzalutamide), which is our primary interest, is likely to remain constant with a constant effect size because of the large number of missing participants. In addition, we can assume that the missingness also occurred in all 12 disease groups in this study, so it is judged to be a non-differential misclassification error, and the direction is not always towards the null value. Therefore, we can maintain confidence in this study."

Q4-2. Analyze AEs by cancer stage or prior treatment lines. Highlight in the introduction stage prostate cnacer analysis prevouis work such as PMID: 30890858 or similar work.

A4-2) We sincerely appreciate your valuable comments. Based on your recommendation, we have added this study in main body as below (line 111-113).

“Recent studies have reported that it is possible to predict the progression of prostate cancer in advance using transcriptomic biomarkers, but it is still not easy to identify side effects using transcriptomic biomarkers.”

Q4-3. Expand on how findings should guide ARPI selection (e.g., avoiding apalutamide in patients with skin conditions).

A4-3) We sincerely appreciate your valuable comments. Based on our results, it is possible to accurately educate patients about the possibility of skin problems before prescribing a specific ALPI, namely apalutamide, and to enable early response and dose adjustment when they occur. In addition, the high side effects of other specific ALPIs can be clinically applied in the same

Q4-4. please highlight the limitation of this work.

A4-4) We sincerely appreciate your valuable comments regarding the limitation of the study. In response to the reviewers' comments, we have included a detailed discussion of the limitations of this study in the text below (line 320-338).

“Our study has several limitations, first, it is a cross-sectional study, so we cannot obtain time dependent information, and we cannot obtain information on the stage and clinical situation of the individual. In addition, reports of AEs may include ADT in combination with or in combination with other chemotherapy regimen, making it difficult to distinguish whether those reported AEs are solely due to ARPIs. Second, even in this FAERS data, there is a significant number of participants who did not report their age and gender, which appears to be a significant bias. However, FAERS is a large big data set and we believe that the PRR for each of the individual groups of ARPI (Enzalutamide, Apalutamide, Darolutamide, Abiraterone, and Abiraterone with Enzalutamide), which is our primary interest, is likely to remain constant with a constant effect size because of the large number of missing participants. In addition, we can assume that the missingness also occurred in all 12 disease groups in this study, so it is judged to be a non-differential misclassification error, and the direction is not always towards the null value. Therefore, we can maintain confidence in this study. Third, the inability to control for potential confounders limits its applicability to real-world practice. Unfortunately, FAERS does not provide detailed exact covariates (comorbidities, concurrent medications, disease stage, etc.) for individual participants, so we tried to control for these as much as possible through stratification by age, gender, and treatment group. The findings should therefore be interpreted as hypothesis-generating and descriptive of reporting trends, rather than definitive comparative risk quantifications as it is primarily stemming from the FAERS data.”

6. PLOS authors have the option to publish the peer review history of their article (what does this mean?). If published, this will include your full peer review and any attached files.

If you choose “no”, your identity will remain anonymous but your review may still be made public.

Do you want your identity to be public for this peer review? For information about this choice, including consent withdrawal, please see our Privacy Policy.

Reviewer #1: Yes: Mohammad Reza Fattahi

Reviewer #2: Yes: Mohammad Ebrahimnezhad

Reviewer #3: No

Reviewer #4: Yes: Abedalrhman Alkhateeb
