# Supplementary material for: Adverse events of androgen receptor pathway inhibitors in prostate cancer from real world data
Source: PLoS One. 2025 Oct 24;20(10):e0335459. doi: 10.1371/journal.pone.0335459 (PMC12551900; doi:10.1371/journal.pone.0335459)
Supplement: Attachment — Submitted filename: Response to Reviewers 250922.docx [file pone.0335459.s012.docx]

PONE-D-25-09399R1

Adverse events of androgen receptor pathway inhibitors in prostate cancer from real world data

PLOS ONE

Dear Dr. SHIM,

Thank you for submitting your manuscript to PLOS ONE. After careful consideration, we feel that it has merit but does not fully meet PLOS ONE’s publication criteria as it currently stands. Therefore, we invite you to submit a revised version of the manuscript that addresses the points raised during the review process.

Please submit your revised manuscript by Sep 25 2025 11:59PM. If you will need more time than this to complete your revisions, please reply to this message or contact the journal office at [plosone@plos.org](mailto:plosone@plos.org). When you're ready to submit your revision, log on to <https://www.editorialmanager.com/pone/> and select the 'Submissions Needing Revision' folder to locate your manuscript file.

Please include the following items when submitting your revised manuscript:

- A rebuttal letter that responds to each point raised by the academic editor and reviewer(s). You should upload this letter as a separate file labeled 'Response to Reviewers'.
- A marked-up copy of your manuscript that highlights changes made to the original version. You should upload this as a separate file labeled 'Revised Manuscript with Track Changes'.
- An unmarked version of your revised paper without tracked changes. You should upload this as a separate file labeled 'Manuscript'.

If you would like to make changes to your financial disclosure, please include your updated statement in your cover letter. Guidelines for resubmitting your figure files are available below the reviewer comments at the end of this letter.

If applicable, we recommend that you deposit your laboratory protocols in [protocols.io](http://protocols.io/) to enhance the reproducibility of your results. Protocols.io assigns your protocol its own identifier (DOI) so that it can be cited independently in the future. For instructions see: [https://journals.plos.org/plosone/s/submission-guidelines#loc-laboratory-protocols](https://track.editorialmanager.com/CL0/https:%2F%2Fjournals.plos.org%2Fplosone%2Fs%2Fsubmission-guidelines%23loc-laboratory-protocols/1/010f0198989d368b-00f26a3e-9baf-4f9a-8c0d-ee82a53c719f-000000/gFmpUGGestLeJEVNYWaZjJTf7NAC3a9qcIufR_cQxU0=223). Additionally, PLOS ONE offers an option for publishing peer-reviewed Lab Protocol articles, which describe protocols hosted on [protocols.io](http://protocols.io/). Read more information on sharing protocols at [https://plos.org/protocols?utm_medium=editorial-email&utm_source=authorletters&utm_campaign=protocols](https://track.editorialmanager.com/CL0/https:%2F%2Fplos.org%2Fprotocols%3Futm_medium=editorial-email%26utm_source=authorletters%26utm_campaign=protocols/1/010f0198989d368b-00f26a3e-9baf-4f9a-8c0d-ee82a53c719f-000000/MkBW85E1Pc8ZFaax_95ZQ3t2s9zW5dIjv9u7Rcbs68E=223).

We look forward to receiving your revised manuscript.

Kind regards,

Xing-Xiong An, M.D.
Academic Editor
PLOS ONE

Journal Requirements:

If the reviewer comments include a recommendation to cite specific previously published works, please review and evaluate these publications to determine whether they are relevant and should be cited. There is no requirement to cite these works unless the editor has indicated otherwise. 

Please review your reference list to ensure that it is complete and correct. If you have cited papers that have been retracted, please include the rationale for doing so in the manuscript text, or remove these references and replace them with relevant current references. Any changes to the reference list should be mentioned in the rebuttal letter that accompanies your revised manuscript. If you need to cite a retracted article, indicate the article’s retracted status in the References list and also include a citation and full reference for the retraction notice.


[Note: HTML markup is below. Please do not edit.]

Reviewers' comments:

Reviewer's Responses to Questions

**Comments to the Author**

1. If the authors have adequately addressed your comments raised in a previous round of review and you feel that this manuscript is now acceptable for publication, you may indicate that here to bypass the “Comments to the Author” section, enter your conflict of interest statement in the “Confidential to Editor” section, and submit your "Accept" recommendation.

Reviewer #1: (No Response)

Reviewer #3: All comments have been addressed

Reviewer #4: All comments have been addressed

2. Is the manuscript technically sound, and do the data support the conclusions?

The manuscript must describe a technically sound piece of scientific research with data that supports the conclusions. Experiments must have been conducted rigorously, with appropriate controls, replication, and sample sizes. The conclusions must be drawn appropriately based on the data presented.

Reviewer #1: Yes

Reviewer #3: Yes

Reviewer #4: Yes

3. Has the statistical analysis been performed appropriately and rigorously?

Reviewer #1: Yes

Reviewer #3: Yes

Reviewer #4: Yes

4. Have the authors made all data underlying the findings in their manuscript fully available?

The [PLOS Data policy](http://track.editorialmanager.com/CL0/http:%2F%2Fwww.plosone.org%2Fstatic%2Fpolicies.action%23sharing/1/010f0198989d368b-00f26a3e-9baf-4f9a-8c0d-ee82a53c719f-000000/22kJVEIdVZIlBqBlhSkB1uXGPMH-oPf6yy9mxNmUedI=223) requires authors to make all data underlying the findings described in their manuscript fully available without restriction, with rare exception (please refer to the Data Availability Statement in the manuscript PDF file). The data should be provided as part of the manuscript or its supporting information, or deposited to a public repository. For example, in addition to summary statistics, the data points behind means, medians and variance measures should be available. If there are restrictions on publicly sharing data—e.g. participant privacy or use of data from a third party—those must be specified.

Reviewer #1: Yes

Reviewer #3: Yes

Reviewer #4: Yes

5. Is the manuscript presented in an intelligible fashion and written in standard English?

PLOS ONE does not copyedit accepted manuscripts, so the language in submitted articles must be clear, correct, and unambiguous. Any typographical or grammatical errors should be corrected at revision, so please note any specific errors here.

Reviewer #1: Yes

Reviewer #3: Yes

Reviewer #4: Yes

6. Review Comments to the Author

Please use the space provided to explain your answers to the questions above. You may also include additional comments for the author, including concerns about dual publication, research ethics, or publication ethics. (Please upload your review as an attachment if it exceeds 20,000 characters)

Reviewer #1: Thank you for the thoughtful revisions and the improvements made to the manuscript. You've addressed several important points, but there are still a few areas that could use a bit more clarification or refinement:

Q1-1) There are a few instances of repetitive content between nearby sentences in both the Introduction and Discussion (e.g., lines 102–105 & 108–111; 309–312 & 316–319). Consider tightening these sections to improve clarity and flow.

A1-1) We sincerely appreciate your valuable comments. In our effort to faithfully address the comments from all reviewers, some sentences became redundant. As you have pointed out, we have revised the manuscript to the best of our ability to correct this.

“The selection of an optimal ARPI for advanced prostate cancer is a critical clinical challenge. Although randomized controlled trial (RCT)s have confirmed the efficacy of various ARPIs, there is a significant lack of comparative data on their adverse effect profiles.” (line 101-103)

“The efficacy of ARPIs in delaying advanced prostate cancer progression is also well-documented by RCTs; however, a gap in evidence regarding their safety profiles challenges optimal treatment selection.” (line 107-109)

“Current knowledge of ARPI side effects relies on RCTs and related meta-analyses. However, significant heterogeneity among these studies leads to inconsistent conclusions about adverse events. Large-scale observational studies using RWD are needed to overcome this limitation, but such research has been absent. Our study is the first to address this gap by analyzing ARPI side effects using a large RWD cohort.” (305-309)

Q1-2) In line 167, "three levels" may be a typo based on the context of the results — consider double-checking this.

A1-2) It’s a typo. 5 level is correct. Thank you very much. (line 165)

Q1-3) I’m a bit unclear about the "All treatments" group in Table S2 — does it refer to all AE reports related to ARPIs (including excluded ones), or to all prostate cancer treatments in general? Based on Figure 1, it seems to be the former. If that's the case, the consistently higher PRRs in the included cases compared to all cases (included + excluded) suggest that patients with a higher burden of AEs were retained. This could challenge the authors’ claim that excluded cases had minimal impact on overall results. Even though PRRs aren’t directly comparable across AE categories, it's notable that groups like “Infection” (PRR: 2.28) appear overrepresented compared to categories like “Endocrine” (PRR: 1.41).

Q1-4) Some clarification would be helpful here.
A brief explanation of how the confidence intervals were calculated (e.g., method or formula) in the Methods section would be helpful for clarity.
A1-3 & 1-4) Thank you for your accurate point. I have already described the formula and result in the method and results. To help the reviewer’s understand related PRR and 95%CI calculating, I will explain it as schematically as possible. If the analysis target group (ARPI groups) changes, the denominator value changes and the overall ratio is recalculated. In addition, if the side effects to be analyzed change (12 disease groups), the numerator value changes and the overall ratio is recalculated as well.

I also attach a table capture so that you can check the calculation yourself. As you know, 95% CI is a basic statistical calculation method that multiplies the standard error by a Z-value of 1.96, so this study does not describe it separately.

Q1-5) It would be helpful to explain the rationale behind the AE classification—beyond the process outlined in Table S1. Was an established or previously used method followed? For instance, categorizing "pneumonia" under Infection rather than Respiratory could be debated, so some clarification on the reasoning would strengthen the approach.

A1-5) Thank you for your careful consideration. It is limited to group all side effect symptoms into exactly the same side effect group. So it is best to group similar groups. As you correctly pointed out, pneumonia can potentially fall into both groups of side effects: infection and respiratory disease.

Therefore, this categorization was classified jointly by clinicians with over 20 years of relevant clinical experience and medical informatics specialists who are experts in standard medical terminology for adverse events. Furthermore, to review any potential misclassifications, conflicts of opinion were resolved through a full research team meeting. The detailed contents of this categorization are described in detail below.

“One author (JHK), a specialist in Urology and prostate cancer, validated the identified terms and their groupings. The authors also analyzed all narrative descriptions of concurrent illnesses and comorbidities in FAERS. In cases of disagreement regarding the descriptions, the final PTs were established through agreement among all investigators.” (line 152-156)

There has always been a problem with categorization issue as it is now, but basically classifying according to MedDRA concepts at the PT level has been proven through a number of previous studies of this author (below Ref 1-7). In particular, Ref 1-3 of the references here classified AE group in exactly the same way.

Ref 1. Kim SJ, Rhee TG, Shim SR. Autoimmune and auto-inflammatory adverse events after COVID-19 vaccination in the United States. Clin Immunol. 2023 Dec 27;259:109882. doi: 10.1016/j.clim.2023.109882. Epub ahead of print. PMID:38158168.

Ref 2. Shin J, Shim SR, Lee J, Ryu HS and Kim J-Y (2024) Otorhinolaryngologic complications after COVID-19 vaccination, vaccine adverse event reporting system (VAERS). Front. Public Health. 11:1338862.

Ref 3. Shim SR, Kim KT, Park E, Pyun JH, Kim JH, Chung BI. Urological complications after COVID 19 vaccine according to age, sex and manufacturer. World J Urol. 2023 Aug;41(8):2255-2263. doi: 10.1007/s00345-023-04481-1.

Ref 4. Kim HJ, Lee SJ, Sa S, Bae JH, Song G, Lee CW, Kim JH, Shim SR, Hong M, Han HW. Safety of COVID-19 Vaccines among Patients with Type 2 Diabetes Mellitus: Real-World Data Analysis. Diabetes Metab J. 2023 May;47(3):356-365. doi:10.4093/dmj.2022.0129.

Ref 5. Song G, Chung SJ, Seo JY, Yang SY, Jin EH, Chung GE, Shim SR, Sa S, Hong MS, Kim KH, Jang E, Lee CW, Bae JH, Han HW. Natural Language Processing for Information Extraction of Gastric Diseases and Its Application in Large-Scale Clinical Research. J Clin Med. 2022 May 24;11(11):2967.

Ref 6. Sa S, Lee CW, Shim SR, Yoo H, Choi J, Kim JH, Lee K, Hong M, Han HW. The Safety of mRNA-1273, BNT162b2 and JNJ-78436735 COVID-19 Vaccines: Safety Monitoring for Adverse Events Using Real-World Data. Vaccines (Basel). 2022 Feb 17;10(2):320.

Ref 7. Lee CW, Sa S, Hong M, Kim J, Shim SR, Han HW. Adverse Events and Safety Profile of the COVID-19 Vaccines in Adolescents: Safety Monitoring for Adverse Events Using Real-World Data. Vaccines (Basel). 2022 May 9;10(5):744.

Q1-6) I noticed that although the use of ARPIs hasn’t declined in recent years, the number of reported AEs has gone down, according to FAERS data. I understand this could be due to many factors, but could changes in treatment strategies—like using lower doses of abiraterone or prescribing ARPIs earlier in the disease—be playing a role? If so, splitting the study period into earlier vs. later years might help clarify whether such shifts are contributing to the trend.

A1-6) We sincerely appreciate your exact comments regarding the confounding factors in FAERS. The changes in treatment strategies pointed out by the reviewer are not well recognized by our authors at the moment. Also, this study looks at the overall trends over the past 10 years, so if we divide them, the number of samples in the case of dalolutamide becomes smaller, as you pointed out in the first review course, so there is a limit to analyzing the study period separately. In the next study, we will make sure to make it an important point of hypothesis setting.

Q1-7) In line 239, the term "prevalence" may not be the most accurate choice—perhaps a more precise term like “reporting frequency” or “proportion of reports” would better reflect the data.

A1-7) Thank you for your accurate point. We revised it to "reporting frequency" (line 237) as you recommended. Thank you again because your careful consideration is increasing the completeness of the study.

Q1-8) Given the nature of the data, it’s important to note that a higher PRR for a specific AE—like skin complications—doesn’t necessarily mean the drug causes more of those events compared to others. I believe this limitation should be acknowledged more clearly in the manuscript.

A1-8) Thank you for valuable consideration. I think there needs to be some change of perspective. PRR is also a risk ratio after all. The PRR of a specific side effect is to compare the rate of occurrence of that drug with the rate of occurrence of the total drug, so if there is a drug PRR in the same side effect, it is comparable to each other. It is possible to compare because it is a standardized value as if RR and OR are compared to each other.

Q1-9) Since the combination of abiraterone and enzalutamide is not a routine practice with established benefits, it would be helpful to clarify why this specific pairing was chosen over other possible combinations. Also, was this use concurrent or sequential? Some explanation would strengthen the rationale.

A1-9) Thank you for valuable consideration. While clinical guidelines do not routinely recommend concurrent use of these two androgen synthesis inhibitors, it was difficult to determine from our data whether patients used these medications simultaneously or sequentially. However, since a substantial number of patients were classified as having received both abiraterone and enzalutamide during their treatment course, we considered it clinically meaningful to analyze this group as a separate cohort to evaluate potential adverse effects and treatment outcomes. Additionally, you can see that Group 5 (abiraterone plus enzalutamide, n = 689) has a sample size similar to Group 3 (darolutamide, n = 733), which is not insignificant and should not be ignored, so it was included in the analysis. This analysis provides valuable real-world insights into the safety profile of patients who have been exposed to both agents, regardless of the specific timing or sequence of administration.

Q1-10) I recommend adjusting for other medications used in combination therapies, such as ADT or docetaxel. Have the authors considered stratifying groups based on whether these agents were co-administered? I understand sample size may be a limitation, but exploring this could add valuable context.

A1-10) We sincerely appreciate your exact comments regarding the combination therapy methods in FAERS. Early in our analysis, we considered the reviewer's methodology. Because it is not presented as individual exact data, but only as aggregate statistics, we established the analysis method while thinking about its use in real practice. Therefore, we first showed the incidence of AEs within individual ARPIs (Table 3) and secondly showed the frequency of adverse events of specific ARPIs in the entire population as PRR (Figure 2). As you mentioned, we did not compare the direct comparisons between ARPIs and combinations, because the FAERS data does not provide precise covariates for individual patients, only aggregate statistics, we felt that analyzing incomplete observational data as a quantitative direct comparison between ARPIs would introduce more bias. Therefore, in the supplemental documents, we present the PRRs stratified by each ARPI group (Enzalutamide, Apalutamide, Darolutamide, Abiraterone, Abiraterone with Enzalutamide), and we have also categorized them by AE or ARPI so that clinicians can differentiate between them when treating patients. The main hypothesis of this study is to compare the occurrence of side effects between individual ARPI drugs, so the comparison with the combination therapy you gave us now is difficult to deal with in-depth in this study. In the next study, we will set it as a major hypothesis and challenge it.

Q1-11) The discussion would benefit from adding more clinically relevant implications and clearer take-home messages for physicians—while still emphasizing appropriate caution given the limitations of the data source. The discussion would be stronger with a clearer acknowledgment of the potential impact of underreporting and overreporting inherent to the FAERS dataset, as this is a key limitation in interpreting the findings.

A1-11) We sincerely appreciate your exact comments regarding the completeness of the study. The limitations and clinical messages requested by the reviewer during the review process, the consideration, and the take a home message are already covered in detail with the discussion.

“Our study has several limitations, first, it is a cross-sectional study, so we cannot obtain time dependent information, and we cannot obtain information on the stage and clinical situation of the individual. In addition, reports of AEs may include ADT in combination with or in combination with other chemotherapy regimen, making it difficult to distinguish whether those reported AEs are solely due to ARPIs. Second, even in this FAERS data, there is a significant number of participants who did not report their age and gender, which appears to be a significant bias. However, FAERS is a large big data set and we believe that the PRR for each of the individual groups of ARPI (Enzalutamide, Apalutamide, Darolutamide, Abiraterone, and Abiraterone with Enzalutamide), which is our primary interest, is likely to remain constant with a constant effect size because of the large number of missing participants. In addition, we can assume that the missingness also occurred in all 12 disease groups in this study, so it is judged to be a non-differential misclassification error, and the direction is not always towards the null value. Therefore, we can maintain confidence in this study. Third, the inability to control for potential confounders limits its applicability to real-world practice. Unfortunately, FAERS does not provide detailed exact covariates (comorbidities, concurrent medications, disease stage, etc.) for individual participants, so we tried to control for these as much as possible through stratification by age, gender, and treatment group. The findings should therefore be interpreted as hypothesis-generating and descriptive of reporting trends, rather than definitive comparative risk quantifications as it is primarily stemming from the FAERS data.

Future research might also explore pharmacogenomic or molecular markers that predispose patients to certain adverse events, enabling even more tailored ARPI therapy. By deepening our understanding of both the benefits and risks of each ARPI in routine practice, we can optimize prostate cancer treatment strategies to improve survival rates and QoL while minimizing harm.” (line 310-333)

Reviewer #3: I thank the authors for their revisions to the article. I recommend that all previously suggested revisions be addressed: ‘‘The adverse effects of treatments applied to patients can directly affect the quality of life. My suggestion to the authors; Literature data on the quality of life of patients receiving ARPI treatment can be briefly commented on in the discussion section.’’

A3) Thank you very much for your valuable assessment. As your recommendation, we adjusted sentence below:

"we can optimize prostate cancer treatment strategies to improve survival rates and QoL while minimizing harm." (in line 332-333)

Reviewer #4: The authors addressed the reviewers' comments. The paper is in a very good shape. I endorse it for publication.

A4) Thank you very much for your valuable assessment. Our authors will strive for the health benefit of mankind and for more health improvement through the discovery of scientific facts. Thank you.

7. PLOS authors have the option to publish the peer review history of their article ([what does this mean?](https://track.editorialmanager.com/CL0/https:%2F%2Fjournals.plos.org%2Fplosone%2Fs%2Feditorial-and-peer-review-process%23loc-peer-review-history/1/010f0198989d368b-00f26a3e-9baf-4f9a-8c0d-ee82a53c719f-000000/9IX6QsOk4zSu9wv6m43htB3FKo24zUv6y8q6WF0Q0-I=223)). If published, this will include your full peer review and any attached files.

If you choose “no”, your identity will remain anonymous but your review may still be made public.

**Do you want your identity to be public for this peer review?** For information about this choice, including consent withdrawal, please see our [Privacy Policy](https://track.editorialmanager.com/CL0/https:%2F%2Fwww.plos.org%2Fprivacy-policy/1/010f0198989d368b-00f26a3e-9baf-4f9a-8c0d-ee82a53c719f-000000/JfoVFQO65PiM17rVdVJOIlnR_XHYPNfItfmjylgdQT8=223).

Reviewer #1: **Yes:** Mohammad Reza Fattahi

Reviewer #3: No

Reviewer #4: **Yes:** Abedalrhman Alkhateeb
